# Severe Life-Threatening Pregnancy Complications, “Near Miss” and Maternal Mortality in a Tertiary Hospital in Southern Nigeria: A Retrospective Study

**DOI:** 10.1155/2020/3697637

**Published:** 2020-07-01

**Authors:** Ubong Bassey Akpan, Udeme Asibong, Ezukwa Omoronyia, Kazeem Arogundade, Thomas Agan, Mabel Ekott

**Affiliations:** ^1^Feto-Maternal Unit, Department of Obstetrics and Gynaecology, University of Calabar, Calabar, Nigeria; ^2^Department of Family Medicine, University of Calabar, Calabar, Nigeria; ^3^Department of Obstetrics and Gynaecology, University of Calabar, Calabar, Nigeria; ^4^Saving Mothers Giving Life Initiative, Pathfinder International, Watertown, MA, USA; ^5^Fertility Unit, Department of Obstetrics and Gynaecology, University of Calabar, Calabar, Nigeria

## Abstract

**Background:**

Investigating severe life-threatening pregnancy complications that women encounter and the maternal morbidities (near miss) may help to evaluate the quality of care in health facility and recommend ways to improve maternal and infant survival especially in low-income countries. The aim of this review was to identify, classify, and determine the frequency and nature of maternal near miss events and the maternal and perinatal outcomes.

**Methods:**

A retrospective facility-based review of cases of near miss and maternal mortality occurring between 1^st^ January 2012 and 31^st^ December 2016 at the University of Calabar Teaching Hospital was conducted. Near miss case definition was based on the WHO disease specific criteria. The main outcomes included the maternal mortality ratio (MMR), maternal near miss ratio (MNMR), mortality index, maternal morbidities, and perinatal outcome.

**Results:**

There were 10,111 pregnancy-related admissions, 790 life-threatening pregnancy complications that resulted in 99 maternal deaths, and 691 near miss cases. The maternal mortality ratio was 979 maternal deaths per 100,000 live births, and the maternal near miss ratio was 6,834 per 100,000 maternities. The MMR to MNMR ratio was 1 : 8. Sepsis and severe anaemia had high case-specific mortality indices of 0.4 and 0.53, respectively. The perinatal outcome was poor compared to that of uncomplicated pregnancies: perinatal mortality rate (PMR) 266 per 1000 live births (OR 7.74); neonatal intensive care (NIC) admissions 11.6 percent (OR 1.83); and low birth weight (LBW) (<2.5 kg) 12.19 percent (OR 1.89).

**Conclusion:**

Antenatal care and early recognition of danger signs in pregnancy as well as prompt referral and early institution of essential obstetrics care are important for maternal and infant survival.

## 1. Introduction

Pregnancy and childbirth are physiological processes that may bring joy to many families and unexpected sorrows and suffering to some families. The tragedy of maternal deaths has generated several discussions in low-income countries, and any woman who is pregnant is at risk of developing complication. Severe acute maternal morbidity or obstetrics “near miss” is an event, in which a pregnant woman comes close to maternal death but does not die—a “near miss” [1].

The World Health Organization (WHO) working group on classification of maternal death defined near miss as “a woman who nearly died but survived a complication that occurred during pregnancy, childbirth, or within 42 days of termination of pregnancy irrespective of the site and the duration” [2]. In most cases, women whose immediate survival is threatened and who survive by chance or because of the hospital care they received may sustain immediate or long-term morbidities.

The observed decline in the maternal mortality ratio globally, especially in the developed nations where it is now a single digit, makes it less effective in evaluating the quality of care in health facilities or maternal audit; hence, near miss data may offer a more useful information in that regard [3–8].

Serious forms of maternal morbidity occur in about 1% of women in the United States compared to 3.01–9.05% in some developing countries [9]. Worldwide, the leading causes of near miss morbidity are haemorrhage and eclampsia/preeclampsia [1, 9]. These complications can have lasting effects, and their sequelae may result in chronic maternal medical disorder including psychosocial problems, injury, and disability [9].

Many maternal deaths as well as related severe morbidities are often reported among women who did not receive antenatal care from skilled birth attendants and usually referred late when they develop life-threatening obstetric complications [10, 11]. In developing countries, with poor obstetric services, emergency intrapartum transfers are very common. Sometimes due to ignorance, poverty, and poor transportation network, outcomes from such cases are usually devastating to the woman, her baby, and the immediate family [11]. These women who are of low-socioeconomic status contribute significantly to the burden of maternal mortality and morbidity [10–12].

Despite massive intervention programmes to improve safe motherhood, maternal mortality and morbidity continue to be high in Nigeria. Most publications on the subject of maternal mortality and “near miss” often focus on the peripartum events and neglect the complications occurring in the early period of pregnancy such as ruptured ectopic pregnancy with massive haemorrhage leading to hypovolemic shock, severe bleeding or sepsis from molar pregnancies, and unsafe abortion as well as indirect causes of maternal death such as severe malaria, AIDS/HIV, and tuberculosis in pregnancy. Hence, we carried out a comprehensive review of pregnancy-related complications in the facility in line with the WHO definition of maternal near miss and mortality criteria.

## 2. Methods

### 2.1. Hospital Setting

The study was conducted in the University of Calabar Teaching Hospital (UCTH), one of the second generation tertiary health institutions located in south-south geopolitical zone of Nigeria. The hospital provides obstetric and gynaecological services to all categories of women, whether registered (booked) for antenatal care or referred. A review of the records revealed that about 180–250 deliveries are conducted monthly at the facility of which 10–15 percent is unbooked or referred for gynaecological or obstetric emergencies.

## 3. Study Design and Identification of Cases

This was a 5-year retrospective review. A thorough review of medical records of women with pregnancy-related complications irrespective of the gestational age managed in the maternity unit of the hospital from 1^st^ January 2012 to 31^st^ December 2016 was conducted, and near miss cases and maternal mortality were identified. The disease specific criteria adopted by Filippi et al. [1] were applied to identify cases. These include severe obstetrics haemorrhage (leading to shock, emergency peripartum hysterectomy, coagulation defects, and/or blood transfusion of 2 or more pints); hypertensive disorders in pregnancy (eclampsia and severe preeclampsia); dystocia (prolonged obstructed labour, uterine rupture, or impending uterine rupture); infection (hyperthermia or hypothermia or a clear source of infection and clinical signs of septic shock); and severe maternal anaemia (low haemoglobin level: haematocrit < 6 g/dl) or clinical signs of severe anaemia in women without severe haemorrhage.

For each case of “near miss” or mortality identified, data were collected on demographic characteristics including parity and gestational age at the time of sustaining the near miss morbidity, nature of obstetric complication(s), treatment received, and maternal and perinatal outcomes. Early pregnancy complications such as sepsis from unsafe abortion, severe haemorrhage from unsafe abortion, and ruptured ectopic pregnancy leading to shock were identified in the gynaecological ward register, and the data were also retrieved and analyzed. Information on maternal deaths and deliveries conducted during the reviewed period were obtained from the delivery registers and case files from the Medical Records Department. Women with missing case files or those who did not continue treatment at the hospital after the initial admission and diagnosis were excluded from the analysis.

## 4. Data Analysis

Data were compiled in a database program, Microsoft Excel, and the statistical analysis was performed. The results data were presented as proportions or percentages. The odds ratio and *p* value were calculated where applicable to test the level of significance.

## 5. Definitions of Terms

### 5.1. Maternal Death

This is the death of a woman while pregnant or within 42 days of termination of pregnancy, irrespective of the duration and site of the pregnancy, from any cause related to or aggravated by the pregnancy or its management but not from accidental or incidental causes.

### 5.2. Severe Acute Maternal Morbidity (Samm or Near Miss)

Acute pregnant or recently delivered woman (within six weeks after termination of pregnancy or delivery) in whom immediate survival is threatened and who survives by chance or because of the hospital care she receives.

### 5.3. Perinatal Period

This commences at 28 completed weeks of gestation and ends seven completed days after birth.

### 5.4. Still Birth

This is death prior to the complete expulsion or extraction from its mother or a fetus/baby of 1000 grams or 28 weeks gestation; the death is indicated by the fact that after such separation, the fetus does not breath or show any other evidence of life, such as beating of the heart, pulsation of the umbilical cord, or definite movement of voluntary muscles.

### 5.5. Perinatal Death

A perinatal death is a fetal death (still birth) or an early neonatal death.

### 5.6. Early Neonatal Death

These are deaths of newborn babies occurring during their first seven days of life.

### 5.7. Peripartum Period

This is the period of pregnancy after the gestational age of viability (28 completed weeks), during delivery, and within 42 days after the delivery period.

## 6. Results

There were 10,111 pregnancy-related admissions during the period of review out of which 790 were identified as life-threatening complications, which resulted in 691 obstetric near miss and 99 maternal deaths.

The crude maternal mortality rate was 9.79 per 1000 pregnancy-related admissions. The calculated maternal near miss rate was 68.34 per 1000 maternities or that is about 6.8 percent of the total pregnancy-related admissions during the period of review. The total number of peripartum admission was 9,894, and the total number of live birth was 9457. The real maternal mortality ratio was 994 per 100,000 live births. More than half of the women with life-threatening complications were in the age range of 20–30 years, and the bulk of them (82.5%) did not receive antenatal care from skilled birth attendants prior to the onset of complication.  Table 1 shows the demography of the women.

Of the 691 maternal “near miss” cases, 358 (45.3%) developed complications before 28 weeks of gestation, while 432 (54.7%) presented with peripartum complications.

Haemorrhage topped the list of maternal near miss events accounting for 298 (43.1%), while anaemia and sepsis carried the highest case-specific fatality rate of 53.3% and 40%, respectively. The mortality index (MI) of sepsis was 0.4 compared to 0.09 for haemorrhage (odds ratio: 4.44) (Table 2). Of the 298 cases of obstetric haemorrhage recorded, complications occurring in the first and second trimesters of pregnancy contributed to 171 (57.4%). One hundred and sixty-three (95.3%) of these were massive intraperitoneal haemorrhage from ruptured ectopic pregnancy, while the rest were bleeding from unsafe abortion and one case of hydatidiform mole. Haemorrhage and sepsis were the major life-threatening presentations of unsafe abortion. Only 3 women died of haemorrhage from ruptured ectopic pregnancies. Most deaths from haemorrhage were due to antepartum or postpartum bleeding. The risk of maternal death from haemorrhage was compared with those of other complications and the odds ratio and is shown in Table 2.

**Figure 1:**
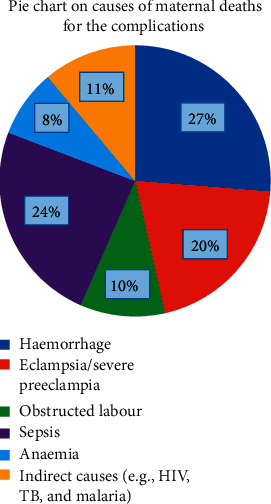
Pie chart showing causes of maternal deaths for the complications.

The various causes of maternal mortality are shown in the pie chart (Figure 1).

In the peripartum morbidities, the most frequent mode of delivery was caesarean section, giving a caesarean section rate of 69.4% among the cases. Spontaneous vaginal deliveries accounted for 23.9%, and laparotomy for uterine rupture was performed in 24 women (5.5%), and instrumental vaginal delivery was performed in about 0.5%. Compared with uncomplicated pregnancies within the period of review, women with near miss events had a significant higher caesarean section rate (69.4% versus 32.1%; odds ratio 2.16).

**Figure 2:**
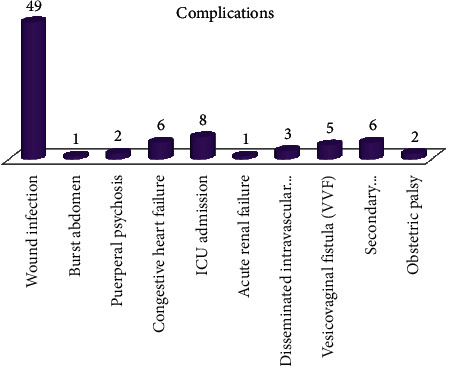
Bar chart showing complications.

Wound infection was the most frequent immediate complication and was recorded in 49 cases. The common long-term complications included vesicovaginal fistula (5) and secondary amenorrhea or infertility (6). Severe uncontrolled obstetric haemorrhage from uterine rupture resulting in peripartum hysterectomy and complications of eclampsia were the main indications for admission to intensive care unit (Figure 2).

Perinatal outcomes were analyzed. Singleton birth was recorded in 422 of the cases, twin birth in 9, and 1 triplet giving a total of 443 babies. Among these women with life-threatening obstetric morbidities, a total of 118 (27.3%) perinatal deaths (stillbirths and early neonatal deaths) were recorded (Table 3). Women with obstetrics complication were significantly more likely to encounter perinatal death compared to women with uncomplicated pregnancies (OR: 7.74; *p* < 0.0001). Uterine rupture was associated with 100% perinatal mortality rate.

**Figure 3:**
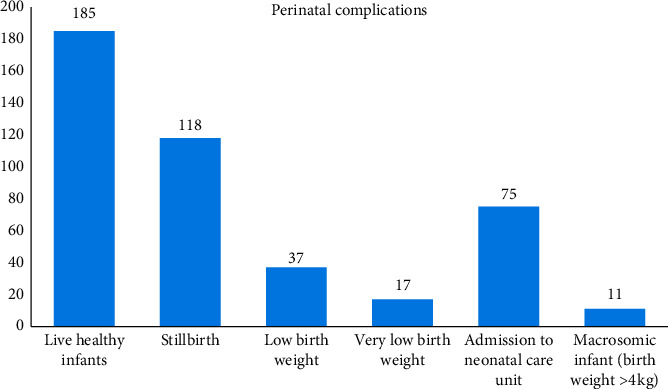
Bar chart showing the perinatal complications.

Live infants in good condition were delivered in 41.5% of the cases of women with “near miss.” About 17% of the babies were admitted to neonatal intensive care unit for treatment (Figure 3).

## 7. Discussion

This study comprehensively reviewed, identified, and classified every patient who was managed life-threatening pregnancy events according to the WHO definition and criteria for near miss and maternal mortality. The maternal near miss rate of approximately 6.8 per 100 admissions recorded in this study is almost similar to the 6.17 per 100 recorded in another study in West Africa [12]. However, it is relatively very high compared to the 1 per 100 live births in the United States of America [9] and 13.8 per 1000 live births in Canada [11]. In the developing countries of West Africa, issues related to ignorance and poverty coupled with superstitious beliefs are factors that often lead to delay in recognizing danger signs and seeking proper and essential care early. This is reflected in this study, which showed that about four-fifths of them did not receive care from skilled birth attendants prior to the onset of presentation in the hospital. Birth preparedness and complication readiness are very vital in the success of safe motherhood initiatives [13]. The maternal death to near miss ratio of approximately 1 : 8 indicates that for every eight women with life-threatening obstetric emergency, one maternal death was recorded. This is relatively lower than the value of 1 : 5 reported in a western Nigerian tertiary hospital [14].

The disparity in the near miss ratio between reports from Teaching Hospitals in West Africa and studies conducted in the industrialized countries may be due to differences in definition and identification of cases regarded as major limitations in comparison of near miss data across institutions [14]. Studies in the industrialized countries commonly use ICU admission or organ system dysfunction/failure as their criteria for case selection. In this study, we used the disease specific criteria adopted by Rosmans and Filippi [2] because it mirrors the major causes of maternal mortality. It also has the advantage of readily comparison that allows assessment of the standard of care with respect to common causes of maternal deaths. A major limitation of using ICU admission for case selection is that it is dependent on factors such as availability of bed space, capacity and location of ICU, and hospital guidelines for ICU admission [14].

The MMR of 994 per 100,000 live births is very high compared to the recent global rate by the WHO in 2015 [15]. More frightening is the finding that more than 50% of them were below 30 years of age. This is a wake-up call for government and international concerned organizations to develop policies that aim toward improving maternal health and enhance women survival. The crude maternal mortality rate instead of ratio was also calculated to compare with the near miss rate. This allowed inclusion of cases occurring in early pregnancy that would not lead to live birth but can result in maternal death or near miss irrespective of the site and the duration of pregnancy according the WHO definition criteria.

The study revealed sepsis as a major killer of women of child bearing ages. These included septic incomplete abortion, puerperal sepsis, and severe surgical wound infections. Late presentation, high cost of drugs, and antibiotic resistance were major factors. Essential obstetric care may not be successful without the availability of essential drugs. There is need to emphasize the necessity of training and retraining of health professionals involved in termination of pregnancies on safe abortion care including the use of manual vacuum aspiration (MVA) to minimize the complications that are associated with unsafe abortions. It is also important for policy makers to include sex education in secondary school curriculum and to provide affordable contraceptives for the teenagers. Poverty has been identified as a major social cause of maternal death in southern Nigeria [15]. Good nutrition as well as women equality and empowerment are possible measures to prevent obstructed labour and its complications.

In a retrospective review of maternal mortality in the same institution by Agan et al. [16], Type I delay, which is the delay in recognizing danger signs of obstetric complications and decision to seek care, was responsible for 35% of maternal deaths. Type I and Type II delay are major problems in sub-Saharan Africa.

Similar to findings in many previous studies, haemorrhage and hypertensive disorders were the leading causes of obstetric near miss accounting for more than 60% of the cases recorded in our study. Although the incidence of severe obstetric haemorrhage was significantly higher, sepsis and eclampsia had a higher case fatality rates compared to obstetric haemorrhage. Judicious use of blood transfusion and availability of misoprostols in the delivery ward for prevention and management of postpartum haemorrhage were effective means of preventing maternal deaths from haemorrhage.

All cases of eclampsia admitted during the reviewed period received at least loading doses of magnesium sulfate. The main causes of death in this group were cerebral haemorrhage, anaesthetic complications, disseminated intravascular coagulopathy, and respiratory failure.

The management of ruptured ectopic pregnancy during the reviewed period deserves commendation as only 3 maternal deaths were recorded out of the 163 cases, in which more than 60% presented with hypovolemic shock. Liberal use of crystalloids, colloids, and blood transfusion including autotransfusion were measures, which effectively reduce maternal deaths. Furthermore, the caesarean section rate was high among women with pregnancy complication due to indications such as severe preeclampsia and eclampsia, obstructed labour, and antepartum haemorrhage.

Severe anaemia in pregnancy leading to heart failure was rare but carried a high case-specific mortality index in this study. A similar finding was reported in another study [14]. The main cause of death in this group was congestive heart failure. And most of these patients arrived at the hospital late when they had developed heart failure. Low social class, poor nutrition, hook worm, and malaria infections as well as haemoglobinopathy are major factors in the tropics.

This study shows that life-threatening obstetric events are potentials for subsequent long-term disability as some of them developed complication such as VVF.

The perinatal outcomes were significantly poor compared to those with uncomplicated pregnancies. Uterine rupture and obstructed labour were major threats to fetal/neonatal survival. Babies delivered to mothers with obstetric near miss were also more likely to require neonatal intensive care due to birth asphyxia, prematurity, and potential for sepsis.

### 7.1. Limitation

A major limitation of this research was its retrospective nature. We could not also assess if survivors of these obstetric complications were satisfied with the quality of care they received while in the hospital. A prospective study which would involve interviewing the women who survived the life-threatening obstetric complications may yield more information on this regard.

## 8. Conclusion

This review had given an insight into the frequency of severe maternal morbidities and mortalities. The high mortality from sepsis, anaemia, and eclampsia suggests that a special unit should be dedicated for management of these conditions. Essential drugs including broad spectrum antibiotics should be provided free to the poor patients in an emergency condition. Blood bank should be sited in the maternity complex, and the public should be enlightened on the need to donate blood to the blood bank. The need for free access to antenatal care where women are educated on birth preparedness and recognition of danger/warning signs of impending obstetric disaster and timely decision on seeking essential obstetric care should form important components of the reproductive health plan in sub-Saharan Africa.

## Figures and Tables

**Table 1:** Sociodemographic features of the women.

	Total, *n* = 790		Percentage
	Near miss, *n* = 691	Mortality, *n* = 99	
Age group			
19 and below	106	15	15.3%
20–29	384	49	54.8%
30–39	169	33	25.6%
40 and above	32	2	4.3%
			100%

Marital status			
Married	547	68	615 (77.8%)
Unmarried	144	31	175 (22.2%)

Parity			
0-1	205	31	236 (29.9%)
2–4	352	42	394 (49.9%)
>4	134	26	160 (20.2%)

Booking			
Status booked	122	16	138 (17.5%)
Unbooked	569	83	652 (82.5%)

**Table 2:** Maternal outcome of obstetrics near miss cases.

	Complication, *n* = 790		Near miss mortality index or	Odds ratio
Diagnosis	Near miss, *n* = 691	Mortality, *n* = 99	case fatality rate per 100 admissions (%)	
Haemorrhage	298	26	8.73	0.97
Eclampsia/severe preeclampsia	180	20	11.1	1.23
Obstetric labour	114	10	8.8	1.00
Sepsis	60	24	40	4.44
Anaemia	15	8	53.3	5.92
Indirect causes	24	11	4.58	0.51
Total	691	99		

**Table 3:** Comparison of perinatal outcomes.

Complication	Near miss infants, *n* = 443	Uncomplicated pregnancy	Odds ratio
Perinatal mortality rate	266.4 per 1000 live births	34.3 per 1000 live births	7.74
Admission to neonatal intensive care	16.93 percent per 100 births	9.23 per 100 births	1.83
Birth weight less than 2.5 kg	12.19 per 100 births	6.45 per 100 births	1.89

## Data Availability

The data used to support the findings of this study are available from the corresponding author upon request.

## Abbreviations

MMR: Maternal mortality rate
MNMR: Maternal near miss rate
MI: Mortality index
PMR: Perinatal mortality rate.
