# Erratum to “Severe Life-Threatening Pregnancy Complications, “Near Miss” and Maternal Mortality in a Tertiary Hospital in Southern Nigeria: A Retrospective Study”

**DOI:** 10.1155/2020/9732648

**Published:** 2020-10-28

**Authors:** Ubong Bassey Akpan, Udeme Asibong, Ezukwa Omoronyia, Kazeem Arogundade, Thomas Agan, Mabel Ekott

**Affiliations:** ^1^Feto-Maternal Unit, Department of Obstetrics and Gynaecology, University of Calabar, Calabar, Nigeria; ^2^Department of Family Medicine, University of Calabar, Calabar, Nigeria; ^3^Department of Obstetrics and Gynaecology, University of Calabar, Calabar, Nigeria; ^4^Saving Mothers Giving Life Initiative, Pathfinder International, Calabar, Nigeria; ^5^Fertility Unit, Department of Obstetrics and Gynaecology, University of Calabar, Calabar, Nigeria

In the article titled “Severe Life-Threatening Pregnancy Complications, “Near Miss” and Maternal Mortality in a Tertiary Hospital in Southern Nigeria: A Retrospective Study” [1], there was an error in affiliation number 4. The correct affiliation for the author Kazeem Arogundade should be “Saving Mothers Giving Life Initiative, Pathfinder International, Nigeria” instead of “Saving Mothers Giving Life Initiative, Pathfinder International, Watertown, MA, USA.” The error was introduced during the production process of the article, and Hindawi apologises for causing this error in the article. The corrected affiliation appears below.

^4^Saving Mothers Giving Life Initiative, Pathfinder International, Calabar, Nigeria
